# Conceptual DFT-Based Computational Peptidology, Pharmacokinetics Study and ADMET Report of the Veraguamides A–G Family of Marine Natural Drugs

**DOI:** 10.3390/md20020097

**Published:** 2022-01-24

**Authors:** Norma Flores-Holguín, Joaquín Ortega-Castro, Juan Frau, Daniel Glossman-Mitnik

**Affiliations:** 1 Laboratorio Virtual NANOCOSMOS, Departamento de Medio Ambiente y Energía, Centro de Investigación en Materiales Avanzados, Chihuahua 31136, Mexico; norma.flores@cimav.edu.mx; 2 Departament de Química, Facultat de Ciènces, Universitat de les Illes Balears, E-07122 Palma de Mallorca, Spain; joaquin.castro@uib.es (J.O.-C.); juan.frau@uib.es (J.F.)

**Keywords:** Veraguamides A–G, computational peptidology, conceptual DFT, KID (Koopmans In DFT), ADMET, computational pharmacokinetics

## Abstract

As a continuation of our research on the chemical reactivity, pharmacokinetics and ADMET properties of cyclopeptides of marine origin with potential therapeutic abilities, in this work our already presented integrated molecular modeling protocol has been used for the study of the chemical reactivity and bioactivity properties of the Veraguamides A–G family of marine natural drugs. This protocol results from the estimation of the conceptual density functional theory (CDFT) chemical reactivity descriptors together with several chemoinformatics tools commonly considered within the process of development of new therapeutic drugs. CP-CDFT is a branch of computational chemistry and molecular modeling dedicated to the study of peptides, and it is a protocol that allows the estimation with great accuracy of the CDFT-based reactivity descriptors and the associated physical and chemical properties, which can aid in determining the ability of the studied peptides to behave as potential useful drugs. Moreover, the superiority of the MN12SX density functional over other long-range corrected density functionals for the prediction of chemical and physical properties in the presence of water as the solvent is clearly demonstrated. The research was supplemented with an investigation of the bioactivity of the molecular systems and their ADMET (absorption, distribution, metabolism, excretion, and toxicity) parameters, as is customary in medicinal chemistry. Some instances of the CDFT-based chemical reactivity descriptors’ capacity to predict the pKas of peptides as well as their potential as AGE inhibitors are also shown.

## 1. Introduction

Understanding the ability of the natural world to produce secondary metabolites is crucial in a number of fields, including medication development. The marine environment is the largest terrestrial ecosystem and prolific producer of biologically active compounds, accounting for more than 70% of the Earth’s surface due to its tremendous richness, which is mostly unexplored compared to terrestrial habitats. Marine habitats contain a large number of unique bioactive compounds with significant therapeutic promise. To cope with abnormal environmental conditions such as oxidative stress, photodynamic damage, and high temperatures, marine animals have been known to produce bioactive chemicals. The variety of bioactive marine compounds with great therapeutic potential is unrivaled, and the chemical and physical conditions of the sea play a role in their development. Many academics are investigating marine natural chemicals with a wide variety of biological activity as a possible source for developing human health-care and management drugs [1,2,3].

Peptides and peptidomimetics are re-emerging as promising possibilities for developing therapeutic solutions for a variety of diseases. Peptides, unlike tiny chemical molecules, have a high degree of specificity, preventing secondary off-target effects, as well as a low level of toxicity. The ability to quickly attach peptides to functionalized nanoparticles, thus enhancing their transport and cellular uptake, provides further benefits [4].

Due to their vast range of applications, excellent biological activity, and great specificity, peptides have surpassed small molecules as medicines in recent decades. However, one of the most significant obstacles to overcome if peptides are to become effective medications is their limited oral bioavailability and physiological instability. Cyclic peptides are important in this context because they are more stable under physiological conditions, have higher membrane permeability, and have higher oral bioavailability than their linear analogues [5,6].

When compared to linear analogues, cyclic peptides frequently have better structural stability and biological activity. Endogenous proteases are less able to degrade this structural rigidity, resulting in improved plasma stability. Peptide cyclization can also help peptides move through cell membranes more easily. As a result, cyclic peptides outperform linear peptides in most ways, including metabolic stability, membrane permeability, and oral bioavailability [7,8].

**Figure 1:**
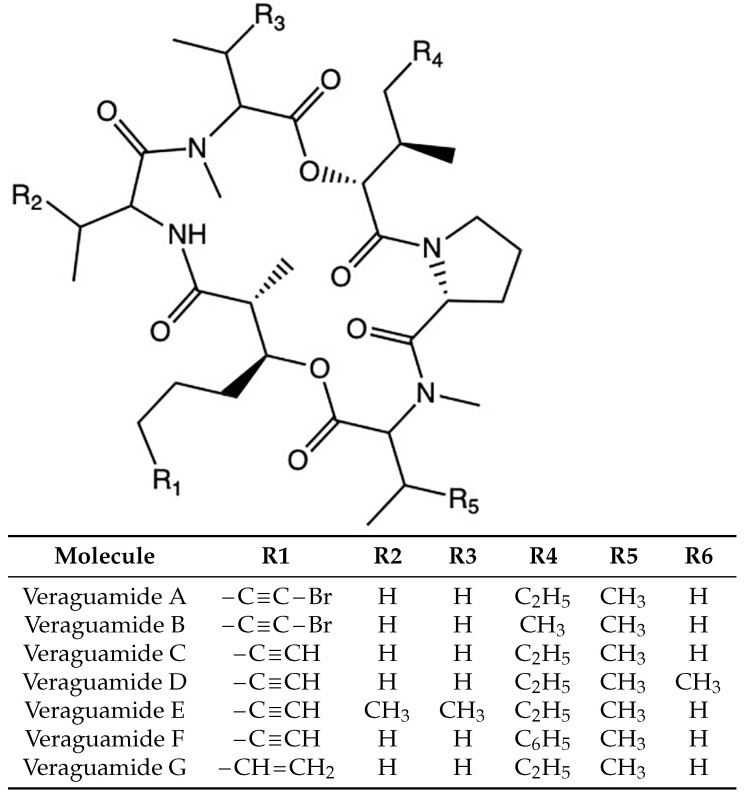
Two-dimensional representations of the molecular structures of the Veraguamides A–G family of marine cyclic hexadepsipeptides.

The study of the chemical, physical and biochemical properties of cyclic peptides of marine origin is of utmost importance because of their potential therapeutic properties. Many families of marine cyclopeptides have been discovered in the last two decades and a lot of effort has been put into the understanding of their bioactivity. One group of these interesting molecules are the Veraguamides A–G, which are cyclic hxadepsipeptides isolated from marine cyanobacteria with potential anticancer properties [9,10,11,12,13,14]. Their two-dimensional structures were retrieved from the PubChem database and are displayed in Figure 1.

As a follow up of our previous studies on the chemical reactivity properties of marine cyclopeptides [15,16,17,18,19], it is worthy of reporting the physicochemical and bioactivity properties of this family of cyclic hexadepsipeptides as well as to predict and understand its chemical reactivity properties considering a methodology developed by our research group. This will be done as a means of further validation of the procedure and for assessing the behavior of different density functionals in the fulfillment of the Janak theorem and the Ionization Energy Theorem, which is a corollary of the former [20,21,22,23,24].

The objective of this work is to report the results of a computational study of the bioactivity properties and chemical reactivity of the members of this family of cyclic peptides on the basis of the CDFT-based computational peptidology (CDFT-CP) methodology [15,16,17,18,19] founded on the combination of the chemical reactivity descriptors from conceptual density functional theory (CDFT) [25,26,27,28,29,30] with some cheminformatics tools [31,32,33,34,35,36,37,38] which may be utilized to assess the associated physicochemical properties to enhance the virtual screening procedure and to detect the ability of the molecules to act as a possible useful drugs, complemented with an analysis of its bioactivity and pharmacokinetics characteristics linked to the ADMET features [39,40,41].

## 2. Methodology

### 2.1. Density Functional Theory (DFT) Calculations

The Kohn-Sham (KS) methodology involves the electronic density, the determination of the molecular energy, and the orbital energies of a specific system, in particular, the HOMO and LUMO frontier orbitals which are intrinsically related to the chemical reactivity of the molecules [42,43,44,45]. The definitions for the global reactivity descriptors are [25,26,27,28,29,30]:

(1) $$\mathrm{Electronegativity}\;\chi \approx \frac{1}{2}\left(\epsilon_{L}+\epsilon_{H}\right)$$

(2) $$\mathrm{Global}\;\mathrm{Hardness}\;\eta \approx (\epsilon_{L}-\epsilon_{H})$$

(3) $$\mathrm{Electrophilicity}\;\omega \approx {\left(\epsilon_{L}+\epsilon_{H}\right)}^{2}/4(\epsilon_{L}-\epsilon_{H})$$

(4) $$\mathrm{Electrodonating}\;\mathrm{Power}\;\omega^{-}\approx {\left(3\epsilon_{H}+\epsilon_{L}\right)}^{2}/16\eta$$

(5) $$\mathrm{Electroaccepting}\;\mathrm{Power}\;\omega^{+}\approx {\left(\epsilon_{H}+3\epsilon_{L}\right)}^{2}/16\eta$$

(6) $$\mathrm{Net}\;\mathrm{Electrophilicity}\;\Delta \omega^{\pm }=\omega^{+}+\omega^{-}$$

being $\epsilon_{H}$ and $\epsilon_{L}$ the frontier orbital energies related to the marine cyclopeptides considered in this research. These global reactivity descriptors that arise from conceptual DFT [25,26,27,28,29,30], has been complemented by the nucleophilicity index N [46,47,48,49,50] that takes into account the value of the HOMO energy obtained by means of the KS scheme using an arbitrary shift of the origin with tetracyanoethylene (TCE) as a reference.

The conformers of the cyclic hexadepsipeptides were established using MarvinView 17.15 from ChemAxon (http://www.chemaxon.com, accessed on 6 October 2021), which was applied in order to undertake molecular mechanics calculations utilizing the MMFF94 force field with an energy window of 0.1 kcal/mol [51,52,53,54,55]. This was followed by a geometry optimization and frequency calculation using the density functional tight binding (DFTBA) methodology [56]. This last step was required for the verification of the absence of imaginary frequencies to confirm the stability of the optimized structure as being a minimum in the energy surface. The determination of the electronic properties and the reactivity descriptors of the Veraguamides A–G addressed the MN12SX/Def2TZVP/H2O model chemistry [57,58,59] because it has been previously shown that it verifies the KID procedure and satisfies the ionization energy theorem, with the aid of the Gaussian 16 software [56] and the SMD solvation model [60]. The charge of all the molecules is taking equal to zero, whereas the radical anion and cation are considered in the doublet spin state. The SMD solvation model was chosen because it has been shown that it provides atomic charges of the Hirshfeld type that are almost independent of the basis set and which are usually recommended for calculations within conceptual density functional theory.

### 2.2. Computational Pharmacokinetics and ADMET Report

The SMILES notation of the cyclopeptides were acquired by accessing the PubChem database, and then were fed into the online program Chemicalize from ChemAxon (http://www.chemaxon.com), which was considered to get a glimpse of the potential therapeutic properties of the studied molecular systems (accessed on 6 October 2021).

A similarity search in the chemical space of compounds with molecular structures that could be compared to the ones being studied, with already known biological and pharmacological properties, was achieved through the online Molinspiration software from Molinspiration Cheminformatics (https://www.molinspiration.com/) (accessed on 6 October 2021). SwissTargetPrediction is an online tool for the prediction of protein targets of small compounds, and it was used to estimate the potential bioactivity of the marine cyclopeptides studied in this research [61].

Pharmacokinetics is a procedure that involves determining the likely fate of a medicinal molecule in the body, which is critical information in the creation of a new medicine. Individual indices named absorption, distribution, metabolism, excretion, and toxicity (ADMET) factors have typically been used to analyze the associated consequences. Chemicalize and the internet-available SwissADME program were used to estimate some ADMET parameters in this study [39]. pkCSM, software for the prediction of small-molecule pharmacokinetic properties using SMILES that can be accessed through its linked webpage, was also used to obtain additional information regarding the pharmacokinetics parameters and ADMET indices [40].

## 3. Results and Discussion

### 3.1. Conceptual DFT-Based Computational Peptidology

The quality of the chosen density function may be realized by comparing its results with results from high-level calculations or from experiential values. Nevertheless, this comparison is not always computationally practicable because of the large size of the molecules or the lack of experimental results for the chemical methods being explored. Our research group has developed a methodology known as KID [15,16,17,18,19] in order to evaluate a particular density functional with regard to its internal coherence. It is evident that within the Generalized Kohn-Sham (GKS) version of DFT, some relationships exist between the KID methodology and the ionization energy theorem, which is a corollary of the Janak theorem [20,21,22,23,24]. This is done by connecting $\epsilon_{H}$ to −I and $\epsilon_{L}$ to −A, through

(7) $$J_{I}=\epsilon_{H}+E_{gs}\left(N-1\right)-E_{gs}\left(N\right)$$

(8) $$J_{A}=\epsilon_{L}+E_{gs}\left(N\right)-E_{gs}\left(N+1\right)$$

(9) $$J_{HL}=\sqrt{{J_{I}}^{2}+{J_{A}}^{2}}$$

Another KID descriptor Δ*SL* related to the difference in energies between the SOMO and the LUMO of the neutral system has been devised to aid in the verification of the accuracy of the methodology. In this work, a new Global KID Descriptor has ben defined as

(10) $$\mathrm{GKD}=\sqrt{{J_{I}}^{2}+{J_{A}}^{2}+{J_{HL}}^{2}+{\Delta SL}^{2}}$$

whose value must be zero for the exact density functional meaning that it verifies the Ionization Energy theorem.

The MN12SX density functional has been shown to have a Koopmans-compliant behavior in earlier peptides studies. However, for a further validation of these model chemistry in the prediction of the chemical reactivity properties of the Veraguamides A–G, additional research is necessary. The CDFT software tool was used to make this determination, and the findings are shown in Table 1, where the GKD has been calculated for all the cyclic peptides using several density functionals and in the presence of solvents of diverse polarity. A recent study [24] has contrasted such behavior with a group of density functionals that includes the usual B3LYP [62,63,64] and PBE0 [65,66] density functionals, the local density functionals BLYP [63,64,67,68] and PBE [69] together with their long-range corrected variants, LC-BLYP and LC-PBE [70], three longe-range corrected density functionals, CAM-B3LYP [71], LC-ωHPBE [72] and ωB97XD [73], as well as three recently proposed density functionals, RSX-PBE, RSX-PBE0 and RSX-PBE0-1/3 [74]. In order to attain completeness, Table 1 shows a comparison of the fulfillment of the ionization energy theorem between the aforementioned density functionals and the screened-exchange MN12SX density functional used in this and previous cyclopeptides research in terms of the dielectric constant ϵ of different common solvents:

The results from Table 1 are very interesting because they show that there are various degrees in the fulfillment of the Janak and ionization energy theorems for the different long-range corrected density functionals involved. Moreover, it can be seen that the agreement varies depending on the dielectric constant of the solvent. Thus, it is possible to see that the CAM-B3LYP and ωB97XD density functionals will be the most accurate when the study of the chemical reactivity is performed in the gas phase, that is, within the absence of any solvent. On the contrary, the MN12SX density functional will be superb when the conceptual DFT chemical reactivity properties estimated in terms of the frontier orbital energies are evaluated in the presence of polar solvents, mainly water, methanol and ethanol. As the chemical and biological processes in which the peptides are involved when they act as therapeutic drugs take part in the presence of water, the results from Table 1 clearly demonstrate the superiority of the MN12SX/Def2TZVP/H2O model chemistry for the study of the molecules like the ones that are being considered through this research.

**Figure 2:**
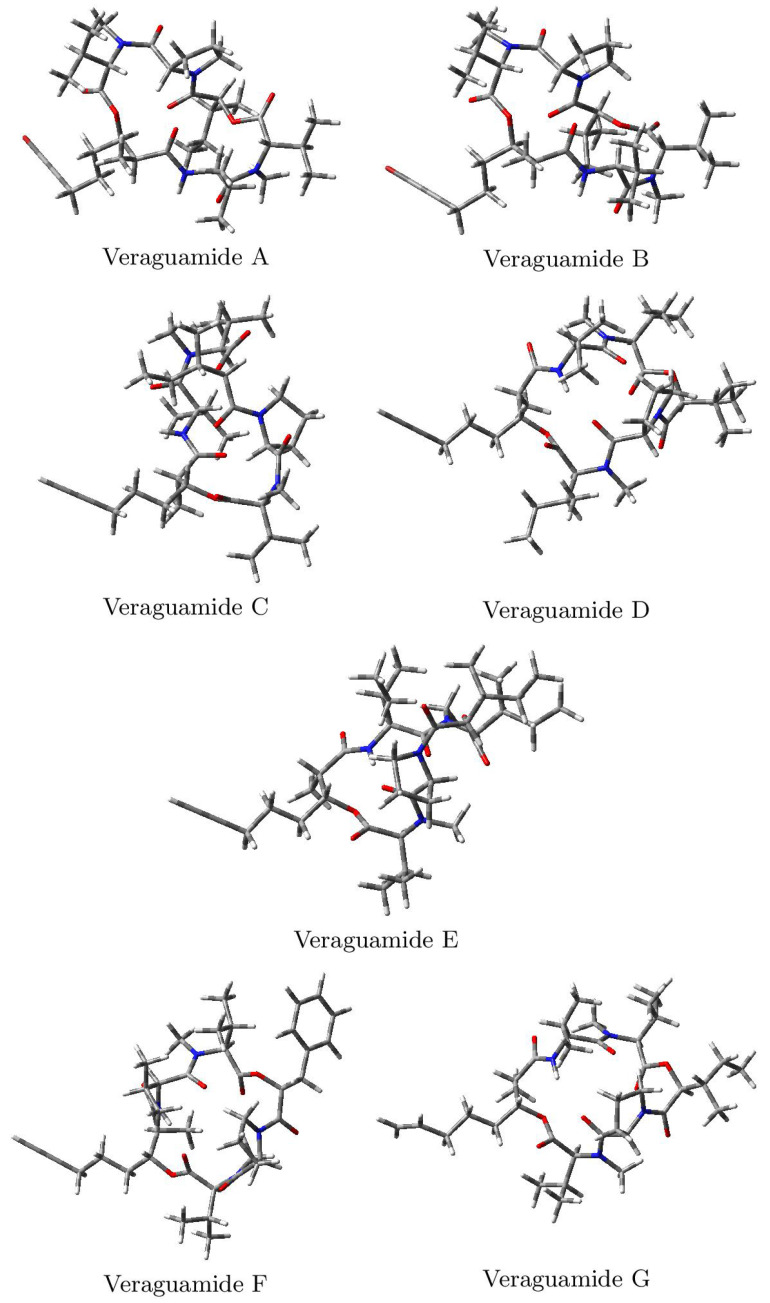
Optimized molecular structures of Veraguamides A–G family of marine cyclic hexadepsipeptides.

The optimized molecular structures of the Veraguamides A–G marine cyclic hexadepsipeptides computed in accordance with the process shown in the Methodology section are displayed in Figure 2:

Having verified that the MN12SX/Def2TZVP is the most adequate one for obtaining accurate results for the conceptual DFT global reactivity descriptors, the values for the frontier orbital energies, the HOMO-LUMO gap and the KID indices are presented in Table 2, while the estimated values for the global reactivity descriptors (including the nucleophilicity N) for the Veraguamides A–G acquired utilizing the mentioned CDFT tool are displayed in Table 3:

The electronegativity (χ) and global hardness (η) are absolute values for the chemical reactivity that have no experimental counterpart. Indeed, they can be estimated by resorting to the experimental vertical ionization energy (I) and vertical electron affinity (A). However, these values are not known for the molecule under study. Going back to the original studies of Robert G Parr and Ralph G Pearson, some kind of classification was done in terms of the HASB principle. This was done only for atoms, ions or very small molecules, for which experimental values for I and A were available at this time. For molecules of the size of the one that we are studying through this research, no standard or experimental values exist. It can only be said something about their global reactivity by comparing with other molecules of the same size. Following this criteria, when comparing with the values of the hardness of some peptides that have been studied recently, it can be said that the veraguamides A–G will be a bit less reactive than those used for comparison because their global hardness values are larger. A different thing can be said about the electrophilicity ω and the nucleophilicity (N). The electrophilicity ω index involves a compromise between the tendency of an electrophile to acquire extra electron density and its resistance to exchange electron density with the environment [50]. By considering a group of Diels-Alder reactions and the electrophiles involved in them [48,75,76], a classification of organic compounds as strong, moderate, or marginal electrophiles, that is, an electrophilicity ω scale, was established, with ω larger than 1.5 eV for the first instance, with ω between 0.8 and 1.5 eV for the second case, and ω smaller than 0.8 eV for the final case [48,75,76]. By checking Table 3, it can be said that all the Veraguamides A–G may be regarded as moderate electrophiles. Domingo and his collaborators [46,47,48,49,50] have also proposed a nucleophilicity index N through the consideration of the HOMO energy obtained through the KS scheme with an arbitrary shift of the origin taking the molecule of tetracyanoethylene (TCE) as a reference. An analysis of a series of common nucleophilic species participating in polar organic reactions allowed them to establish a further classification of organic molecules as strong nucleophiles with N > 3.0 eV, moderate nucleophiles with 2.0 < N < 3.0 eV and marginal nucleophiles with N < 2.0 eV. As seen in Table 3, it can be concluded that, with the exception of veraguamide C and veraguamide E, all the other peptides may be considered as moderate nucleophiles.

**Figure 3:**
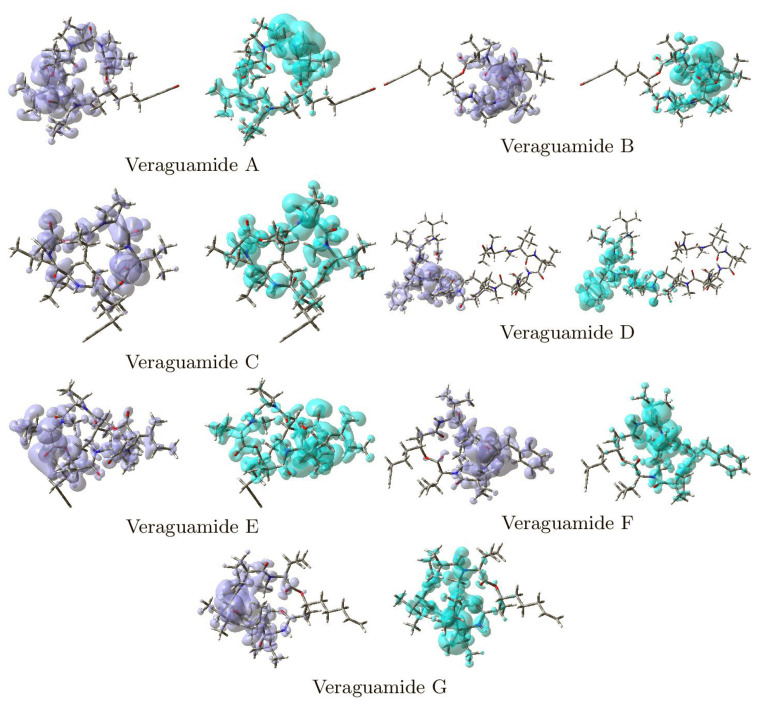
Graphical representation of the dual descriptor DD of the Veraguamide A, Veraguamide B, Veraguamide C, Veraguamide D, Veraguamide E, Veraguamide F and Veraguamide G molecules. **Left**: DD > 0, **Right**: DD < 0.

The global descriptors demonstrate the chemical reactivity of a each molecule in its entirety; therefore, local reactivity descriptors have been designed to assess the differences in the chemical reactivity between the areas inside a molecule. The nucleophilic and electrophilic fukui functions (NFF and EFF) [25,26,27] and the dual descriptor DD [77,78,79,80,81,82] are some of these local reactivity descriptors. They have been defined as: NFF = $\rho_{N+1}\left(r\right)-\rho_{N}\left(r\right)$, EFF = $\rho_{N}\left(r\right)-\rho_{N-1}\left(r\right)$ and DD = ${\left(\partial f(r)/\partial N\right)}_{\upsilon (r)}$, establishing links between the electronic densities of the various species as well as between the NFF and EFF. The NFF identifies molecular locations that are more vulnerable to nucleophilic attacks, whereas the EFF identifies regions that are more vulnerable to electrophilic attacks. The reactive locations have been successfully identified using these local reactivity characteristics. However, the dual descriptor DD has been discovered to be capable of describing both nucleophilic and electrophilic locations within a molecule without ambiguity [82]. Figure 3 shows graphical sketches of the Dual Descriptor DD for the Veraguamides A–G, highlighting the locations where DD > 0 and DD < 0 for a better understanding of these molecules’ local chemical reactivity.

### 3.2. Computational Pharmacokinetics and ADMET Report

**Figure 4:**
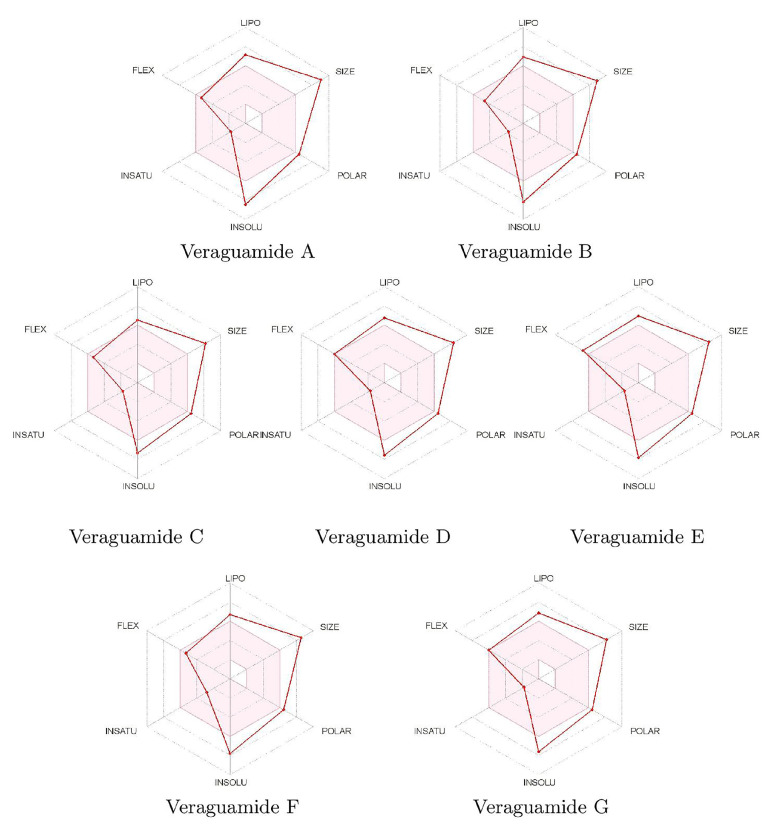
Bioavailability radars of the Veraguamide A, Veraguamide B, Veraguamide C, Veraguamide D, Veraguamide E, Veraguamide F and Veraguamide G molecules.

On the basis of the methodology presented previously, the pKas of the peptides have been estimated following a simple QSAR relationship pKa = 16.3088 − 0.8268η that we have derived during the study of amino acids and small peptides and which has been useful in the study of larger peptides as well as being of interest for the development of advanced glycation end products (AGEs) inhibitors [83]. These results are reported in Table 4:

A compact depiction of the characteristics of the molecules related to their bioavailability can be displayed in a graphical mode through the so-called bioavailability radars displayed in Figure 4 for the veraguamides A–G family of marine cyclic peptides:

It follows that the main difficulties for the veraguamides A–G marine cyclopeptides to be considered as therapeutic drugs of wide bioavailability are those related to their sizes and lack of solubility, and to some extent to their polarities, whose values are somewhat larger than the ideal ones.

The majority of medicinal drugs work by attaching to target protein molecules while at the same time modifying their functions. The Bioactivity Scores, which are a measure of a capacity of the molecules to act or coordinate with distinct receptors, are listed in Table 5 for the veraguamides A–G.

These bioactivity scores for organic molecules can be interpreted as active (when the bioactivity score is greater than 0), moderately active (when the bioactivity score lies between −5.0 and 0.0) and inactive (when the bioactivity score is lower than −5.0).

The main conclusion from the results of Table 5 is that the veraguamides A–G will exert their abilities as therapeutic drugs mainly behaving as protease inhibitors, and to a lesser extent, as GPCR ligands and enzyme inhibitors.

The pharmacokinetics of a drug is evaluated through ADMET research, which is an acronym for absorption, distribution, metabolism, excretion, and toxicity. If absorption is unsatisfactory, the distribution and metabolism of the drug would be changed, potentially resulting in nephrotoxicity and neurotoxicity. As a result, ADMET analysis is one of the most important aspects of computational drug design. In addition to the previous conceptual DFT-based computational peptidology and pharmacokinetics results, we are supplementing this study with a report of the computed ADMET features as shown in Table 6:

It is important to note that all the members of the veraguamides A–G family of cyclopeptides display positive values for the human gastrointestinal absorption (HI) and blood-brain barrier (BBB) permeability and negative values for the AMES toxicity, while the opposite is related to hepatotoxicity. All the peptides will be P-glycoprotein inhibitors (P-gp), being also P-gp substrates. None of the peptides will be inhibitors of the molecules related to cytochrome P450, while all of them will act as substrates of the CYP3A4 variant. Finally, all the cyclic peptides considered here will display a negative result regarding their behavior as hERG inhibitors.

## 4. Conclusions

The chemical reactivity of the veraguamides A–G have been thoroughly investigated by optimizing their structures using DFTBA methodology and calculating their electronic properties using a high-quality model chemistry, namely MN12SX/Def2TZVP/H2O. This model chemistry has already been used in previous research, demonstrating its utility for these types of calculations. However, an involved estimation of a newly-defined KID descriptor for all the peptides with several long-range correct density functionals and in the presence of various solvents of diverse polarity has definitively demonstrated that the MN12SX is superior to other functionals like CAM-B3LYP and ωB97XD when the calculations are performed in the presence of water. The superiority of the MN12SX density functional allowed the estimation of the frontier orbital energies with great accuracy based on the KID procedure evaluation. The fact that the energy of the LUMO and of the SOMO (or the HOMO energy of the anion) are almost the same, which is reflected in the KID accuracy descriptor ΔSL being very close to zero, is an indication that the derivative discontinuity is negligible for the chosen density functional. This is translated as the ability of the LUMO energy to reflect with precision the electron affinity of the molecule, implying that the chemical reactivity parameters obtained by considering this density functional will be very accurate. This is a very important result because it allowed the estimation of the accuracy of the calculation based only on the fulfillment of some intrinsic requirements (like the Janak and ionization energies) without the need to resort to the comparison with experimental results that could not be available, as in the present case.

By considering our suggested Conceptual DFT-based computational peptidology methodology, the seven members of the veraguamides A–G family of cyclic peptides isolated from marine sources have been studied by applying certain methods generally used in the procedure of drug discovery and development, showing that these hexadepsipeptides may be regarded as potential therapeutic drugs. The biological targets, physicochemical attributes, and ADMET indices associated with their bioavailability and pharmacokinetics were forecasted and analyzed as descriptors that could be useful in future QSAR and peptidomimetics studies.

## Figures and Tables

**Table 1:** Average GDK values of the seven members of the Veraguamides A–G family of marine peptides considering several density functionals and different common solvents.

Solvent	1/ϵ	LC-BLYP	LC-PBE	MN12SX	CAM-B3LYP	LC-ωHPBE
NMF	0.0055	4.17	4.16	0.02	2.15	3.88
Formamide	0.0092	4.18	4.17	0.02	2.16	3.89
H2O	0.0128	4.16	4.16	0.01	2.15	3.87
Methanol	0.0307	4.09	4.08	0.05	2.08	3.80
Ethanol	0.0402	4.00	3.99	0.11	2.00	3.81
Acetone	0.0488	3.73	3.72	0.27	1.77	3.45
DCE	0.0988	3.62	3.61	0.37	1.65	3.33
THF	0.1347	3.45	3.44	0.48	1.50	3.16
DBE	0.3282	2.95	2.94	0.85	1.03	2.67
Cyclohexane	0.4959	2.56	2.55	1.14	0.66	2.29
n-Hexane	0.5314	2.48	2.47	2.03	0.58	2.21
Gas	1.0000	1.36	1.35	2.08	0.48	1.09
**Solvent**	**1/** ϵ	ω **B97XD**	**M11**	**RSX-PBE**	**RSX-PBE0**	**RSX-PBE0-1/3**
NMF	0.0055	2.91	3.54	4.06	4.24	4.32
Formamide	0.0092	2.92	3.55	4.08	4.25	4.33
H2O	0.0128	2.90	3.53	4.06	4.23	4.32
Methanol	0.0307	2.84	3.46	3.99	4.16	4.24
Ethanol	0.0402	2.76	3.38	3.38	4.07	4.16
Acetone	0.0488	2.53	3.12	3.01	3.80	3.88
DCE	0.0988	2.42	3.01	2.89	3.68	3.77
THF	0.1347	2.26	2.84	2.72	3.51	3.59
DBE	0.3282	1.80	2.36	2.60	3.02	3.10
Cyclohexane	0.4959	1.43	1.98	2.47	2.63	2.71
n-Hexane	0.5314	1.35	1.90	2.39	2.55	2.63
Gas	1.0000	0.28	0.79	1.26	1.42	1.50

**Table 2:** Frontier orbital energies, H-L gap and the KID indices (all in eV) used for the verification of the ionization energy theorem behavior of the MN12SX density functional in the study of the chemical reactivity of the veraguamides A–G marine natural drugs.

Molecule	HOMO	LUMO	SOMO	H-L Gap	J(I)	J(A)	J(HL)	ΔSL
Veraguamide A	−6.635	−0.884	−0.892	5.751	0.006	0.003	0.006	0.003
Veraguamide B	−6.637	−0.942	−0.912	5.695	0.008	0.012	0.014	0.030
Veraguamide C	−6.812	−0.993	−0.946	5.819	0.003	0.017	0.017	0.048
Veraguamide D	−6.661	−0.748	−0.759	5.913	0.010	0.005	0.011	0.011
Veraguamide E	−6.873	−0.717	−0.702	6.156	0.002	0.007	0.007	0.014
Veraguamide F	−6.717	−1.016	−0.972	5.702	0.002	0.015	0.015	0.019
Veraguamide G	−6.697	−0.771	−0.768	5.926	0.009	0.001	0.009	0.003

**Table 3:** Global reactivity descriptors for the veraguamides A–G family of marine cyclopeptides: Electronegativity (χ), Hardness (η), Electrophilicity (ω) (all in eV), Softness S (in ${\mathrm{eV}}^{-1}$), Nucleophilicity N, Electrodonating Power ($\omega^{-}$), Electroaccepting Power ($\omega^{+}$) and Net Electrophilicity ($\Delta \omega^{\pm }$) (also in eV).

Molecule	χ	η	ω	S	N	$\omega^{-}$	$\omega^{+}$	$\Delta \omega^{\pm }$
Veraguamide A	3.760	5.751	1.229	0.174	2.157	4.697	0.937	5.634
Veraguamide B	3.790	5.695	1.261	0.176	2.156	4.772	0.983	5.755
Veraguamide C	3.903	5.819	1.309	0.172	1.981	4.932	1.030	5.962
Veraguamide D	3.704	5.913	1.160	0.169	2.132	4.543	0.838	5.381
Veraguamide E	3.795	6.156	1.170	0.162	1.920	4.621	0.827	5.448
Veraguamide F	3.867	5.702	1.311	0.175	2.075	4.912	1.045	5.957
Veraguamide G	3.734	5.926	1.177	0.169	2.096	4.590	0.856	5.447

**Table 4:** Predicted pKas for the Veraguamides A–G family of marine cyclopeptides.

Molecule	pKa
Veraguamide A	12.36
Veraguamide B	12.40
Veraguamide C	12.55
Veraguamide D	12.58
Veraguamide E	12.60
Veraguamide F	12.50
Veraguamide G	12.62

**Table 5:** Bioactivity Scores of the veraguamides A–G.

Molecule	GPCR	Ion Channel	Nuclear Receptor	Kinase	Protease	Enzyme
	Ligand	Modulator	Ligand	Inhibitor	Inhibitor	Inhibitor
Veraguamide A	−0.42	−1.45	−1.21	−1.18	0.05	−0.83
Veraguamide B	−0.33	−1.31	−1.07	−1.02	0.13	−0.73
Veraguamide C	−0.29	−1.21	−1.06	−0.96	0.20	−0.62
Veraguamide D	−0.42	−1.39	−1.22	−1.18	0.12	−0.76
Veraguamide E	−0.55	−1.57	−1.38	−1.35	0.02	−0.91
Veraguamide F	−0.64	−1.78	−1.55	−1.43	−0.09	−1.09
Veraguamide G	−0.35	−1.32	−1.12	−1.04	0.13	−0.72

**Table 6:** Computed ADMET Features of the Veraguamides A–G.

Property	Veraguamides						
	A	B	C	D	E	F	G
HI Absorption	+	+	+	+	+	+	+
BBB Permeability	+	+	+	+	+	+	+
Caco-2	-	-	-	-	-	-	-
P-gp Substrate	+	+	+	+	+	+	+
P-gp Inhibitor	+	+	+	+	+	+	+
CYP2C9 Substrate	-	-	-	-	-	+	-
CYP2D6 Substrate	-	-	-	-	-	-	-
CYP3A4 Substrate	+	+	+	+	+	+	+
CYP1A2 Inhibitor	-	-	-	-	-	-	-
CYP2C19 Inhibitor	-	-	-	-	-	-	-
CYP2C9 Inhibitor	-	-	-	-	-	-	-
CYP2D6 Inhibitor	-	-	-	-	-	-	-
CYP3A4 Inhibitor	-	-	-	-	-	-	-
OCT2 Substrate	-	-	-	-	-	-	-
AMES Toxicity	-	-	-	-	-	-	-
hERG Inhibitor	-	-	-	-	-	-	-
Hepatotoxicity	+	+	+	+	+	+	+
Skin Sensitization	-	-	-	-	-	-	-

## Data Availability

All data generated from this research is available from the authors under request.
